# Predicting the Types of Ion Channel-Targeted Conotoxins Based on AVC-SVM Model

**DOI:** 10.1155/2017/2929807

**Published:** 2017-04-09

**Authors:** Wang Xianfang, Wang Junmei, Wang Xiaolei, Zhang Yue

**Affiliations:** School of Computer and Information Engineering, Henan Normal University, Xinxiang 453007, China

## Abstract

The conotoxin proteins are disulfide-rich small peptides. Predicting the types of ion channel-targeted conotoxins has great value in the treatment of chronic diseases, epilepsy, and cardiovascular diseases. To solve the problem of information redundancy existing when using current methods, a new model is presented to predict the types of ion channel-targeted conotoxins based on AVC (Analysis of Variance and Correlation) and SVM (Support Vector Machine). First, the *F* value is used to measure the significance level of the feature for the result, and the attribute with smaller *F* value is filtered by rough selection. Secondly, redundancy degree is calculated by Pearson Correlation Coefficient. And the threshold is set to filter attributes with weak independence to get the result of the refinement. Finally, SVM is used to predict the types of ion channel-targeted conotoxins. The experimental results show the proposed AVC-SVM model reaches an overall accuracy of 91.98%, an average accuracy of 92.17%, and the total number of parameters of 68. The proposed model provides highly useful information for further experimental research. The prediction model will be accessed free of charge at our web server.

## 1. Introduction

Conotoxins proteins have many merits, such as low relative molecular mass, stable structure, remarkable activity, high selectivity, and ease of synthesis [1]. Besides, conotoxins have a wide range of applications in the scope of disease treatment, which includes chronic pain, movement disorders, cramps, cancer, and stroke [2]. According to its different targets acting on the organism, the conotoxins can be divided into three categories [3]: (1) acting on voltage-gated ion channels, (2) acting on the ligand-gated ion channel, and (3) acting on other receptors. Further, the voltage-gated ion channels, also known as voltage-sensitive channels, include potassium ion channels, calcium ion channels, and sodium ion channels.

The performance of using different machine learning algorithms in predicting different targets is different. In 2014, neural network and SVM classifier were used to predict lipid binding proteins by Bakhtiarizadeh et al. [4]; the experiments showed that SVM was more successful at discriminating between LBPs and non-LBPs than neural network. In 2016, the potential druggable proteins were predicted through comparing 6 kinds of machine learning algorithms by Jamali et al.; the experiments showed that neural network was the best classifier when predicting potential druggable proteins [5]. In this paper, we will compare the performance of several different machine learning algorithms in the prediction of ion channel types of conotoxin.

There are studies on the prediction of superfamily and family of conotoxins based on protein sequence. In 2006, SVM model was built to predict the superfamily conotoxins based on PseAAC (pseudo amino acid composition) with an overall accuracy of 88.1% by Mondal et al. [6]. In 2007, an IDQD model was proposed based on dipeptide combinations to predict superfamily and family of conotoxins with accuracy of 87.7% and 72%, respectively, by Lin and Li [2]. However, there are few researches on the prediction of ion channel types of conotoxins. In 2011, a feature selection approach based ANOVA was used to predict the types of ion channel [7]. In 2013, an RBF model based on the feature selection method of Binomial Distribution was used to predict the ion channels of three types of conotoxins with an overall accuracy of 89.3% and total of parameters of 70 by Yuan et al. [8]. However, these feature extraction methods belong to winding method, which not only depends on the performance of classifier, but also causes time consumption.

In view of the above problems in the prediction of ion channel types of conotoxins, a model named AVC-SVM is proposed based on AVC and SVM in this paper. First, the *F* value is used to measure the level of significance of all features to the results. Besides, rough selection is carried out to delete the attributes which have less influence on the classification results. Secondly, Pearson Correlation Coefficient [9, 10] is introduced to measure the redundancy among the attributes. Then, threshold is set to filter the features whose correlation is too strong. Finally, SVM was used as a classifier to predict the ion channel types of conotoxins. And results of prediction are used to calculate the sensitivity, average precision, and overall accuracy. Results of 5-fold cross-validation show that the AVC-SVM model has better performance when considering accuracy, the total number of features, and running time as a whole.

## 2. Preprocessing of Data Sets

The data sets used in this experiment were derived from Universal Protein Resource (UniProt). In order to obtain a reliable benchmark database, the following steps are performed according to the literature [8]:

1. Protein sequences must be annotated and evaluated manually.
2. Protein sequences, which contain ambiguous amino acid residues (such as X, B, and Z), should be excluded.
3. Amino acid sequences belonging to other protein fragments should be excluded.
4. Homologous proteins should be excluded.

We used 112 protein sequences as the basic data set which include 24 potassium ion channel-targeted conotoxins, 43 sodium ion channel-targeted conotoxins, and 45 calcium ion channel-targeted conotoxins from [8]. It is necessary to express the protein sequences with the eigenvector of the same number of dimension before predicting [11]. However, the information contained in the eigenvectors tends to be redundant. In the prediction of the ion channel types, the feature selection will directly affect the performance of the classifier [12]. Consequently, it is significant for feature extraction.

## 3. Feature Extraction

The prediction for ion channel types of the conotoxins requires that the protein sequences are represented by the eigenvectors of the same number of dimension. However, there is still redundancy by using general methods of representation of the information. It not only affects the speed of calculation but also affects the results of classification. Therefore, we need to choose the remarkable characteristics of both independence and recognition ability. At present, many feature selection techniques are used to optimize the feature sets, such as ReliefF [13], ReCorre [14], Binomial Distribution [8], and ANOVA [11]. However, few feature selection algorithms have both good prediction accuracy and short running time. In this paper, a novel feature extraction algorithm named AVC is designed to reduce redundancy of attributes and improve the accuracy and speed of prediction.

### 3.1. Features Representation of Protein Sequences

Both amino acid combinations and dipeptide combinations are often used as parameters for feature selection. The dipeptides combination can not only reflect the information of amino acid residues but also reflect the amino acid sequence number information [7]. Parameters of features by dipeptides combination can reflect the information from protein sequence more comprehensively [2], so we selected dipeptide combinations as parameters to represent features of protein sequences. The total number of dipeptides is 400; therefore, there are 400 features. The protein sequence *P* is defined as follows:

(1) $$\begin{array}{c} P=\left[\;a_{1},a_{2},\ldots ,a_{u},\ldots ,a_{400}\;\right], \end{array}$$

where *a*_*u*_ is the frequency of occurrence of the *u*th dipeptide combination in the protein sequence *P*. The calculation method is shown as follows:

(2) $$\begin{array}{c} a_{u}=\frac{X_{u}}{\sum_{u}X_{u}}. \end{array}$$

In (2), *X*_*u*_ is the *u*th dipeptide in the protein sequence.

**Figure 1:**
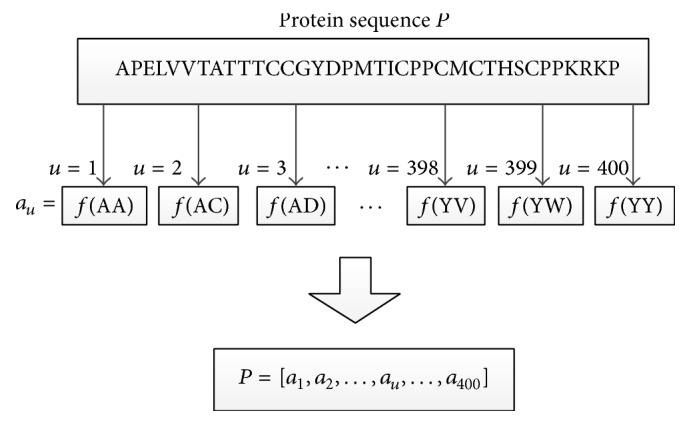
Transferring the raw protein sequence to 400 features.

Here, we take the protein sequence APELVVTATTTCCGYDPMTICPPCMCTHSCPPKRK as an example; the conversion process is shown in Figure 1.

According to the order of the 20 amino acid residues in the alphabet, we arranged 400 dipeptides. When *u* = 1, *a*_1_ = *f*(AA). *f*(AA) counts the frequency of occurrence of the dipeptide AA in the protein sequence sample *P*. Similarly, the frequencies of the emergence of 400 dipeptides are obtained from the proteins sequence sample. Finally, the eigenvectors of each protein sequence are decided.

### 3.2. AVC

The process of the AVC method is described as follows. Firstly, variance-based analysis is used to calculate the ratio *F* of the variance between groups and variance within the group for each attribute [15]. The size of the *F* value is used to measure the recognition capability of the attributes [16]. The larger the *F* value is, the stronger the recognition capability of attribute is [17]. And then the features which have less impact on the results of classification are deleted. Secondly, we introduce Pearson Correlation Coefficient [9, 10] to measure the redundancy of attributes. Threshold is set to filter the features whose correlation is too strong. The *F* value of the *u*th dipeptide is calculated as follows:

(3) $$\begin{array}{c} F\left(\;u\;\right)=\frac{S_{b}^{2}\left(u\right)}{S_{w}^{2}\left(u\right)}, \end{array}$$

where *S*_*b*_^2^(*u*) represents the variance between groups and *S*_*w*_^2^(*u*) represents the variance within groups [18]. The calculation methods are shown in (4) and (5), respectively [19]:

(4) Sb2u=SSbuK−1,

(5) Sw2u=SSwuN−K,

where *K* is the total of classes and *N* is the total of samples. Here, the value of *K* is 3 and the value of *N* is 112. *SS*_*b*_(*u*) is the sum of the squares between the groups. And *SS*_*w*_(*u*) is the sum of squares within the groups [20]. The calculation methods are shown in (6) and (7), respectively:

(6) SSbu=∑i=1Kmi∑j=1miaui,jmi−∑i=1K∑j=1miaui,j∑i=1Kmi2,

(7) SSwu=∑i=1 K∑j=1miaui,j−∑j=1miaui,jmi2,

where *m*_*i*_ denotes the total of samples in the *i*th group (here *m*_1_ = 24, *m*_2_ = 45, and *m*_3_ = 43). *a*_*u*_(*i*, *j*) represents the frequency of the *u*th dipeptide of *j*th samples in the *i*th group. Take the threshold *f*. If *F*(*u*) < *f*, remove *p*(*u*) from all samples. Then the rough selection of attributes is completed. The attribute that is not important to the classification result is deleted, and the new feature matrix *P*_*x*_ is obtained.

Method of variance-based analysis preserves attributes which have strong recognition ability. However, redundancy may exist in the attributes which have strong recognition ability. It is not conducive to the results of prediction. To solve this problem, Pearson Correlation Coefficient is used to measure correlation between attributes [9]. Its value is between −1 and 1 [10]. We can obtain correlation coefficient between dipeptides. The calculation method is shown as follows:

(8) $$\begin{array}{c} r_{uv}=\frac{\sum_{i=1}^{N}\left(a_{u}\left(i\right)-\bar{a_{u}}\right)\left(a_{v}\left(i\right)-\bar{a_{v}}\right)}{\left(N-1\right)S_{a_{u}}S_{a_{v}}}, \end{array}$$

where *a*_*u*_(*i*) represents occurrence frequency of the *u*th dipeptide in the *i*th sample in whole dataset. Similarly, *a*_*v*_(*i*) represents the frequency of occurrence of the *v*th dipeptide of the *i*th sample in whole dataset. $\bar{a_{u}}$ and $\bar{a_{v}}$ are the average of the occurrence frequency of the *u*th dipeptide and the *v*th dipeptide in whole dataset, respectively. *S*_*a*_*u*__ and *S*_*a*_*v*__ are the standard deviation of *a*_*u*_ and *a*_*v*_, respectively. The calculation method of *S*_*a*_*u*__ is shown as follows:

(9) $$\begin{array}{c} S_{a_{u}}=\sqrt{\frac{\sum_{i=1}^{N}{\left(a_{u}\left(i\right)-\bar{a_{u}}\right)}^{2}}{N-1}}. \end{array}$$

The obtained *r*_*uv*_ is compared with a preset threshold *r*_0_. If *r*_*uv*_ > *r*_0_, the correlation between the *v*th attribute and the *u*th attribute is larger than the expected value. It means that there is much redundancy between them. And then we compare the *F* value of the *u*th with *F* value of the *v*th attribute. The attribute whose *F* value is smaller than another is deleted. We can obtain a collection of attributes which are both strong and independent until all attributes are traversed. A new feature matrix *P*_*y*_ is obtained.

## 4. Prediction Principle of AVC-SVM

After feature selection, we need to select an appropriate algorithm to predict the types of ion channels of conotoxins. SVM is a machine learning algorithm based on statistical analysis [21]. It has great advantages in solving nonlinear, small sample and high-dimensional pattern recognition based on the principle of minimizing structural risk [22]. In addition, SVM algorithm also has many applications in bioinformatics [4, 21, 22]. In this paper, the SVM algorithm was used to predict ion channel types of the conotoxins.

**Figure 2:**
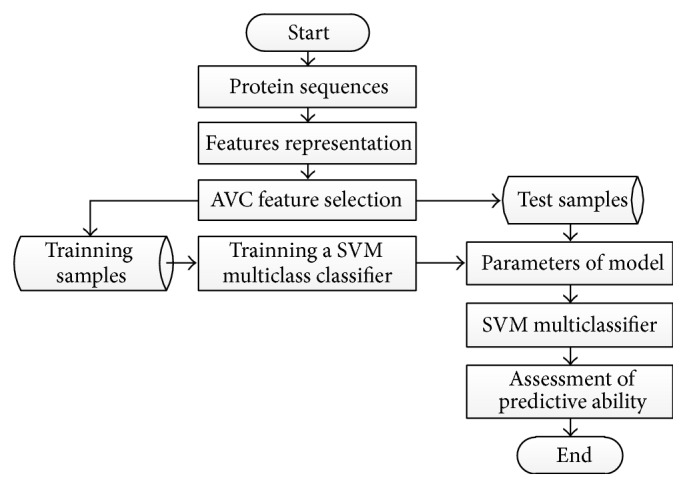
The flow chart for prediction of ion channel types of conotoxins by AVC-SVM model.

The samples are divided into three categories in this paper. Therefore the method of SVM multiclassification is used to predict the ion channel types of conotoxins. There are many methods of SVM multiclassification such as OVR (one-versus-rest), OVO (one-versus-one), and DAG (Directed Acyclic Graph) [23]. We select OVO method to construct a multiclass classifier to predict the ion channel types of conotoxins. The predictive process using AVC-SVM model is shown in Figure 2.

The principle of method of OVO [24] multiclassification is depicted that there are *k*(*k* − 1)/2 classifiers for *k* classes. A classifier is trained for two classes. When classifying an unknown sample, each classifier determines its class and “votes” for the corresponding category. Finally, the category with the largest number of votes is the category of the unknown sample.

### 4.1. Evaluation Criteria

In the study for the prediction of protein function, the evaluation criteria which are widely used are sensitivity (Sn), overall accuracy (OA), and average accuracy (AA) [25]. They are defined as follows:

(10) Sni=TPiTPi+FNi,

(11) OA=∑i=1nTPiN,

(12) AA=∑i=1nSnin,

where TP_*i*_ and FN_*i*_ denote true positives and false positives for the *i*th class, respectively. *N* and *n* denote the total of samples and the total of classes, respectively.

### 4.2. Steps for Prediction

There are five steps to predict the types of ion channels.

**Step 1 .**

Formulae (1) and (2) are used to preprocess the date sets and obtain the feature representation of amino acid sequences.

**Step 2 .**

The *F* value calculated by (5) is used to measure the recognition ability of all attributes. Set the threshold *f*. If *F*(*u*) < *f*, the *u*th attribute value *a*_*u*_ is deleted from all attributes of samples. And, then, a new vector *P*_*x*_ is obtained.

**Step 3 .**

Formulae (8) and (9) are used to calculate the correlation coefficient *r*_*uv*_ between the *u*th attribute and the *v*th attribute in feature matrix *P*_*x*_. Set the threshold *r*_0_; if *r*_*uv*_ > *r*_0_, *F* value of the *u*th attribute is compared with *F* value of the *v*th attribute. Then the attribute whose *F* value is smaller is deleted from the two features.

**Step 4 .**

The 112 samples are divided into 5 subsets randomly. One of the five subsets takes turns as test set; the rest are training set. SVM multiclass method was used to train and predict types of ion channel.

**Step 5 .**

Formulae (10)–(12) are used to evaluate sensitivity, the overall accuracy, and average accuracy of the model.

## 5. Results and Analysis

### 5.1. Results of Attributes Reduction Using AVC

**Figure 3:**
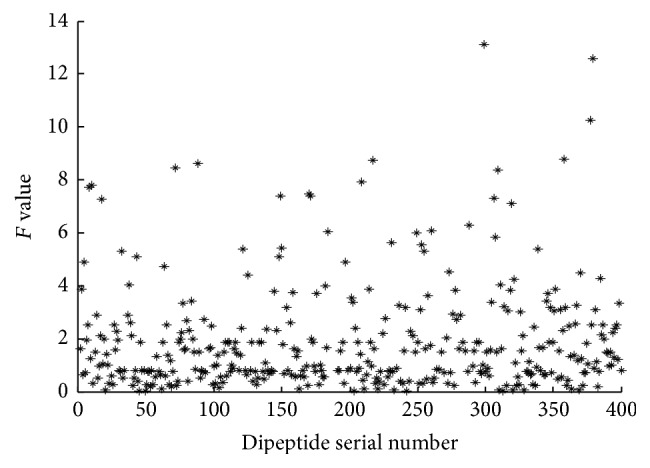
Scatter plot of *F* values for all dipeptides before feature selection.

**Figure 4:**
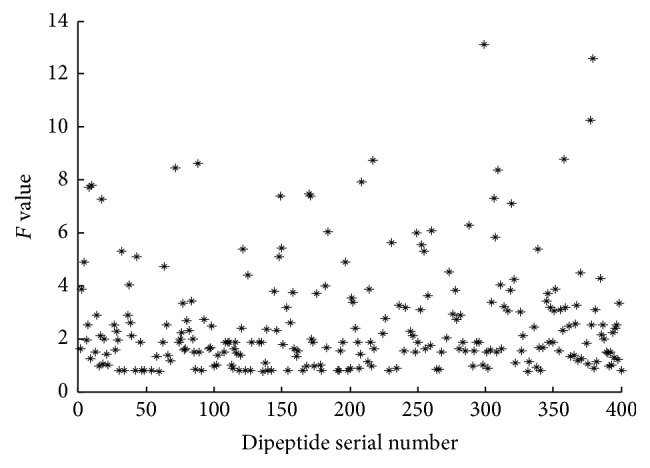
Scatter plot of the *F* value distribution for the portion dipeptides after rough selection.

**Figure 5:**
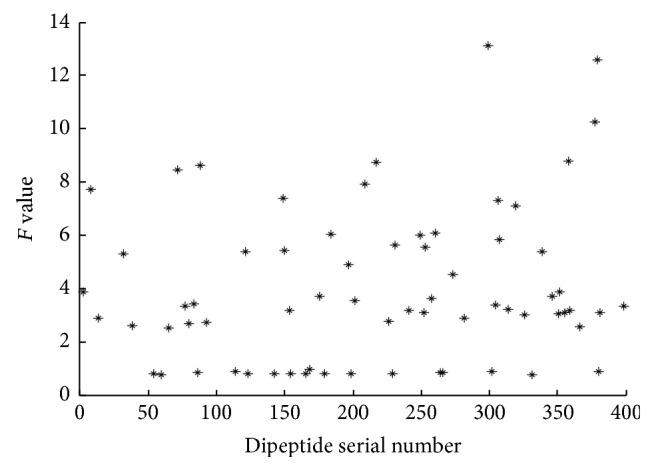
Scatter plot of *F* values for the portion dipeptides after correlation analysis.

The analysis of variance is used to calculate the *F* values of all the attributes. The distribution of *F* value of 400 dipeptides is shown in Figure 3. Figures 4 and 5 are the *F* values of some dipeptides after the rough selection and after the correlation analysis, respectively.

As we can see from Figures 3 and 4, the number of the small *F* values in Figure 3 is less than that in Figure 4. Because the *F* value measures the ability to identify the attribute, the features which have smaller *F* value have less effect on the result. Consequently, these attributes are deleted from all features.  Figure 5 shows the *F* value distribution for the portion dipeptides after correlation analysis. The splashes in Figure 5 become few and sparser than the splashes distributed in Figure 4.  Figure 5 not only shows the features which have the smaller *F* value are deleted but also shows that the features having a strong correlation are deleted. It proves that the method of AVC feature selection can reduce the number of dimensions effectively.

### 5.2. Contrastive Results Using Different Methods for Feature Selection

To further illustrate the effectiveness of our method, Table 1 shows the results of comparison of AVC and different feature selection methods. All the classification algorithms in Table 1 use the SVM method and perform 5-fold cross-validation.

In Table 1, Sn indicates the sensitivities of three types of ion channels. OA is the overall accuracy. And AA is the average accuracy. The accuracy and sensitivity of the AVC, ANOVA (Analysis of Variance), BiDi (Binomial Distribution) [8], ReliefF [26–28], and ReCorre [14] algorithms are compared when using SVM. The AVC method with an average accuracy of 92.17% and an overall accuracy of 91.98% is higher than other methods in Table 1. In addition, the sensitivities in predicting K and Na ion channels using the AVC-SVM method are the highest and reach 93.14% and 94.17%, respectively. The sensitivity using ANOVA method in predicting Ca ion channel is the best and reaches 92.54%. Comparing the principle of AVC, ANOVA, BiDi, and ReliefF, we can find that only AVC can distinguish the redundant features with strong correlation. Comparing the principle of AVC, ReliefF, and ReCorre, we can find that ReCorre algorithm adds the analysis of relativity analysis based on ReliefF but it does not solve the problem of instability caused by noise and exception points. However, the process of weight calculation based on analysis of variance used in this paper has better robustness. In order to compare the efficiency of feature selection, Table 2 shows running time and the resulting dimensions when using different methods of feature selection. The classification algorithm uses SVM uniformly in Table 2.

The results in Table 2 show the running time of AVC-SVM is the shortest and reaches 0.085 s. The running times of ANOVA-SVM, BiDi-SVM, ReliefF-SVM, and ReCorre-SVM are 9.350 s, 11.939 s, 9.478 s, and 7.547 s, respectively. The method with the least dimensions is AVC-SVM with the dimensions of 68.

### 5.3. Comparison Using Different Multiclassification Algorithms

For the choice of classification algorithm, this paper uses SVM algorithm, which is suitable for the prediction of small sample data [4]. Besides, SVM algorithm does not involve the use of probability measure and law of large numbers, so it is different from the existing statistical methods [29]. In order to prove the superiority of SVM in accuracy and sensitivity, further experiments are needed. When using AVC method to feature selection, the comparisons using different prediction algorithms are shown in Table 3. To make the results more reliable, 5-fold cross-validation was used in all the methods in Table 3.

The results show that AVC-SVM is superior to other methods with the highest average accuracy of 92.17% and the highest overall accuracy of 91.98%, respectively. The overall accuracies of Bayes [32], ELM (extreme learning machine) [33], RF (Random Forest) [34, 35], and RBF (radial basis function neural network) [36] are 82.61%, 78.70%, 76.80%, and 66.09%, respectively. Moreover, the sensitivities for the three types of ion channels predicted by SVM are the highest. Comparing SVM with Bayes, ELM, RF, and RBF neural networks, the results show that SVM is the best prediction method when using feature selection of AVC.

### 5.4. Comparison Using Different Models

In recent years, there are some studies on the prediction of ion channel types of conotoxins. The contrast experiments were shown in Table 4.

It can be seen from Table 4 that AVC-SVM model is better than the BiDi-RBF model and iCTX-Type model in terms of average accuracy, overall accuracy, and time efficiency. When compared with *F*-score-SVM, the average accuracy and the overall accuracy of the AVC-SVM model are not as high as those in literature [31]. However, the sensitivity of the AVC-SVM model is better than that of the *F*-score-SVM in predicting K ion channel. Moreover, the number of features and running time used by the AVC-SVM model is less than the *F*-score-SVM model.

The *F* value used in our method and *F*-score proposed by the literature [30] are different. The *F*-score in the literature [30] is the ratio of the variance between groups and the variance within groups. The variance between groups in the literature [30] is calculated using sum of squares of deviations. The *F* value in our paper is the ratio of the mean square deviation between groups and the mean square deviation within groups. In this paper, the mean square deviation is the sum of squares of deviations divided by degree of freedom. It can eliminate the impact caused by imbalance of number of samples between groups.

## 6. Conclusions

In this paper, the proposed model based on feature selection of AVC and prediction method of SVM is used to predict the type of ion channels. The results of 5-fold cross-validation show that our model reaches high predicted accuracies and the feature selection method in this paper has two advantages over other feature selection methods: first, the analysis of correlation for features is used to further reduce the existing information redundancy between the strong correlating features. Second, the calculated process for weights of the attributes is robust. However, it is necessary to declare the data set which is mined for analysis. We will further expand the data set in the follow-up work for in-depth analysis.

## Figures and Tables

**Table 1:** Results of comparison of different feature selection methods.

Methods	Sn^K^ (%)	Sn^Ca^ (%)	Sn^Na^ (%)	AA (%)	OA (%)
AVC-SVM	93.14	89.21	94.17	92.17	91.98
ANOVA-SVM	89.28	92.54	87.79	89.87	89.25
BiDi-SVM [8]	83.3	83.7	93.3	86.8	87.5
ReliefF-SVM	87.11	85.55	76.61	83.08	82.25
ReCorre-SVM	78.67	73.38	82.62	78.22	77.71

**Table 2:** Results of efficiency comparison using different feature selection methods.

Methods	Running time (s)	Dimensions
AVC-SVM	0.085	68
ANOVA-SVM	9.350	163
BiDi-SVM	11.939	167
ReliefF-SVM	9.478	304
ReCorre-SVM	7.547	99

**Table 3:** Results of comparison using different prediction algorithms.

Methods	Sn^K^ (%)	Sn^Ca^ (%)	Sn^Na^ (%)	AA (%)	OA (%)
AVC-SVM	93.14	89.21	94.17	92.17	91.98
AVC-Bayes	66.67	88.89	81.82	79.12	82.61
AVC-ELM	59.05	79.00	90.22	76.09	78.70
AVC-RF	75.95	79.27	79.33	78.19	76.80
AVC-RBF	64.67	59.91	73.59	66.05	66.09

**Table 4:** Results of comparison using different models.

Methods	Sn^K^ (%)	Sn^Ca^ (%)	Sn^Na^ (%)	AA (%)	OA (%)	Dimensions	Running time (s)
AVC-SVM	93.1	89.2	94.2	92.2	92.0	68	0.085
BiDi-RBF [8]	91.7	88.4	88.9	89.7	89.3	70	11.258
iCTX-Type [30]	83.3	97.8	89.8	90.3	91.1	50	8.743
*F*-score-SVM [31]	91.7	95.3	95.6	94.2	94.6	180	10.594
